# Age- and refraction-related changes in anterior segment anatomical structures measured by swept-source anterior segment OCT

**DOI:** 10.1371/journal.pone.0240110

**Published:** 2020-10-23

**Authors:** Xiaobin Xie, Giulia Corradetti, Abe Song, Anmol Pardeshi, William Sultan, Jong Yeon Lee, Fei Yu, Lixia Zhang, Shuang Chen, Vikas Chopra, Srinivas R. Sadda, Benjamin Xu, Alex S. Huang

**Affiliations:** 1 Eye Hospital of China Academy of Chinese Medical Sciences, Beijing, China; 2 Doheny Eye Institute and Stein Eye Institute, Department of Ophthalmology, David Geffen School of Medicine, University of California, Los Angeles, California, United States of America; 3 Roski Eye Institute, Department of Ophthalmology, University of Southern California, Los Angeles, California, United States of America; 4 Department of Ophthalmology, College of Medicine, Gil Medical Center, Gachon University, Incheon, Korea; Bascom Palmer Eye Institute, UNITED STATES

## Abstract

**Purpose:**

To assess the effects of age and refractive status on anterior segment anatomical structures, including the ciliary body, using a new swept-source anterior segment optical coherence tomography (AS-OCT) device.

**Methods:**

This prospective observational study included 63 healthy volunteers (mean age: 44.2 years). Images of the anterior segment were obtained using a new swept-source AS-OCT (ANTERION, Heidelberg Engineering GmbH, Heidelberg, Germany) with tracking and image averaging from the right eye of all participants. Repeatability as well as inter- and intra-observer reliability of biometric measurements were evaluated. The impact of image tracking and averaging on ciliary muscle measurements was tested. Univariate and multivariable statistical models were developed to evaluate the relationship of age and refractive status on anterior segment biometric measurements.

**Results:**

For all test-retest repeatability and inter- and intra-observer reproducibility of swept-source AS-OCT measurements, high intraclass correlation (ICC) was noted (0.88–1.00). The nasal maximum ciliary muscle thickness (CMTMAX) and distance between scleral spur to the thickest point of the ciliary muscle (SSMAX) were larger than those on the temporal side (*p*<0.001 and *p* = 0.006, respectively). Nasal and temporal CMTMAX (*p* = 0.004 and *p*<0.001, respectively) and lens thickness (*p*<0.01) increased with age. Nasal and temporal SSMAX decreased with older age and increasing hyperopia (*p* = 0.01 and *p*<0.001, respectively). Image averaging resulted in improved ciliary muscle measurements (*p =* 0.008 to 0.02). Lens vault increased with older age and increased hyperopia (*p*<0.01). OCT measurements of the angle decreased with older age and increased hyperopia (*p<*0.001 to 0.03). Aqueous depth decreased with older age and increased hyperopia (*p*<0.01). Pupil diameter decreased with older age (*p*<0.01).

**Conclusions:**

Repeatability and reproducibility of biometric measurements using the ANTERION AS-OCT were excellent. Image averaging improved the accuracy of ciliary muscle measurements. The device produced measurements of biometric parameters that described superficial and deep structures including the ciliary body and full lens thickness from a single image.

## Introduction

Optical coherence tomography (OCT) was described over 20 years ago [1], now with wide-spread use. Clinically, posterior segment OCT is nearly standard-of-care for every patient presenting with the posterior-segment disease around the macula and nerve. For research, posterior segment OCT measurements are also highly repeatable so that they can be considered advantageous to functional visual end-points during FDA-trials of age-related macular degeneration and inherited retinal diseases [2]. Recently, AS-OCT has been developed for clinical purposes [3, 4]. It is widely used for research but less so during clinical care (compared to posterior-segment OCT) as not every patient with suspected anterior segment alteration necessarily receives AS-OCT. Limitations of AS-OCT include the absence of image tracking for image averaging and longitudinal follow-up, and the need to better capture the entire anterior segment in one image.

Tracking refers to the process by which OCTs can monitor the position or orientation of the eye based on a reference point or structure to ensure that OCT B-scans are acquired from the same location across or within imaging sessions [5]. High-quality tracking tracking has two important benefits. First, accurate tracking allows longitudinal OCT assessment in the same location in a single subject over time across multiple imaging sessions [6]. This is critical for uncovering the natural history of disease or treatment success/failure over time. Second, tracking within an image acquisition session allows B-scans to be accurately taken in specific locations multiple times [7, 8]. This is important for new imaging modalities such as OCT-angiography which visualizes changes in OCT reflectivity in focal areas [9, 10]. Doing so also allows multiple B-scans from one location to be averaged, which improves image signal-to-noise ratio. At this time, no commercial AS-OCT is known to perform all of these above functions.

In addition, it is difficult to obtain high resolution AS-OCT images that display the entire anterior segment, from the anterior corneal to the posterior lens surface. Imaging the entire anterior chamber is challenging due to its area and depth. However, this is important because the anterior chamber has many anatomical components that contribute to various diseases. As a comparison, posterior segment OCT usually images a macular area not much larger than a few square millimeters and several hundred microns deep. The anterior chamber around the limbus covers a large area (the distance around limbus [2πR]: > 35 mm; surface area [πR^2^]: > 100 square millimeters; and depth of imaging: ~8 mm [from the anterior cornea to the posterior lens]). Based upon commercially available lasers at the time of development, initial time-domain OCTs scanned at a wavelength of ~1300 nm which provided adequate depth of imaging (including the ciliary muscle) but lacked in resolution and speed [4], required to image a large area. Subsequent spectral-domain OCTs used ~800 nm laser wavelengths which improved speed and resolution but then usually focused the image to one side of the eye while not being able to image deep structures such as the ciliary muscle [4]. Swept-source OCT (SS-OCT), using a ~1300 nm laser, improved on this with better speed and depth of penetration [4]. Multiple publications have described SS-OCT for imaging the cornea, anterior chamber, anterior chamber angle, and iris [11–17], although tracking capabilities and full lens thickness imaging could not be performed. Thus, SS-OCT has some inherent advantages for AS-OCT due to its speed and image coverage for large areas at a wavelength which provides more detailed information about ocular anatomy.

Here, we describe a new swept source AS-OCT (ANTERION; Heidelberg Engineering GmbH, Heidelberg, Germany) which provides the theoretical benefits of fast image acquisition speed and adequate depth of imaging. This allows for the visualization of the ciliary muscle and entire lens thickness. Further, this device also has image tracking to allow for image averaging. Overall, images of the entire anterior segment (cornea, anterior chamber angle, iris, lens [portions not directly blocked by the iris], and ciliary muscle) that includes two sides of the eye can be simultaneously acquired in a single B-scan. Therefore, we assessed the repeatability and reproducibility of this device. We tested the impact of image averaging. The ciliary muscle was selected as it has been more difficult to image by OCT technology. Furthermore, multiple anterior segment parameters were evaluated from single images using this device and used to assess anterior-segment anatomical relationships across age- and refractive-groups.

## Methods

### Study design

This prospective observational study included 63 healthy and phakic volunteers (27 females and 36 males) between 26–74 years of age (median [quartiles]; 41 [32–57]) between June 2019 and November 2019. All participants were recruited from normal/healthy volunteers at the Doheny Eye Centers, University of California, Los Angeles (UCLA). This study followed the tenets of the Declaration of Helsinki and was approved by the University of California, Los Angeles Institutional Review Board (#19–000621). Prior to imaging, all participants signed informed consent.

Inclusion criteria comprised of: a complete visual examination within the last three months of being enrolled, having no known pathology, and having best-corrected distance Snellen visual acuity of least 20/25 in both eyes. Exclusion criteria included individuals who had ocular trauma or surgery, those who were <20 years of age, or pregnancy. Only the right eye per participant was included because of the similarity between both eyes in a healthy population [18]. All subjects underwent a complete ophthalmic examination which included visual acuity, refraction, slit-lamp biomicroscopy, and an undilated fundus examination. Objective refraction was obtained, starting with a KR-1W Wave-Front Analyzer (Topcon Group, Tokyo, Japan), and subjective refraction was subsequently determined using a digital optotype chart monitor (Cal Coast 20/20 Acuity System, Torrance, CA, USA). Contact lens users were instructed to refrain from wearing lenses one day prior and during anterior segment imaging to minimize optical surface aberrations.

### Anterior segment OCT imaging protocol

All images were obtained using a new anterior segment OCT (ANTERION, Heidelberg Engineering GmbH, Heidelberg, Germany; software version 1.2) using the Metrics Application. An internal systems test was performed each day when starting the device, according to manufacturer instruction. All images were obtained by a single trained ophthalmologist (XX) following a standardized imaging protocol using the Metrics Application. Dim ambient lighting conditions were strictly maintained during the whole image acquisition procedure because anterior segment structures can change as a result of alterations to ambient illumination [19, 20]. The ambient illumination was measured as 0.02 lux (Dr. Meter Digital Illuminance Meter, model-LX1330B, California, United States) at the imaging plane. Subjects were positioned in front of the instrument with their head placed in a combined head and chin rest. During image acquisition, subjects were instructed to fixate on an internal fixation target in primary gaze while the other eye was covered by an occluder equipped on the device. To image the eye under a relaxed state without accommodation, the refractive correction (spherical equivalent) was inserted into the device using an internal lens system that ranged from -15D to +15D in ± 0.5 D increments. During the image acquisition, subjects were asked to refrain from blinking and to fixate on the instrument’s internal fixation target. The participants were asked to blink before proceeding to the next examination step. Immediately after image acquisition was completed, examination quality was assessed. This was done automatically by in-built software which grades the image based on the following endpoints: possible blinking, motion, fixation, tear film irregularity, refraction correction, tracking, and camera imaging segmentation. If a result of “pass” was not obtained for any of the above patterns, the acquisition was repeated. The acquisition parameters for the swept source AS-OCT device were wavelength: 1300μm; A-scan rate: 50,000Hz; resolution in tissue: <10 microns axially × 30 microns laterally; scan pattern: radial scan; number of A-scans per B-scan: 768; scan length: 16.5 mm and scan depth: 14.5±0.5 mm. The images were tracked using the cornea light reflex, centered on the corneal vertex, to allow for image averaging (6 B-scans averaged).

To determine the repeatability of the anterior OCT imaging, 40 of 63 eyes were imaged twice on separate occasions, 5 minutes apart. The volunteers were asked to sit back and then reposition their chin and forehead between acquisition sessions. The device was shut down and restarted before the second acquisition.

### Anterior eye segment image post-processing and data analysis

**Fig 1:**
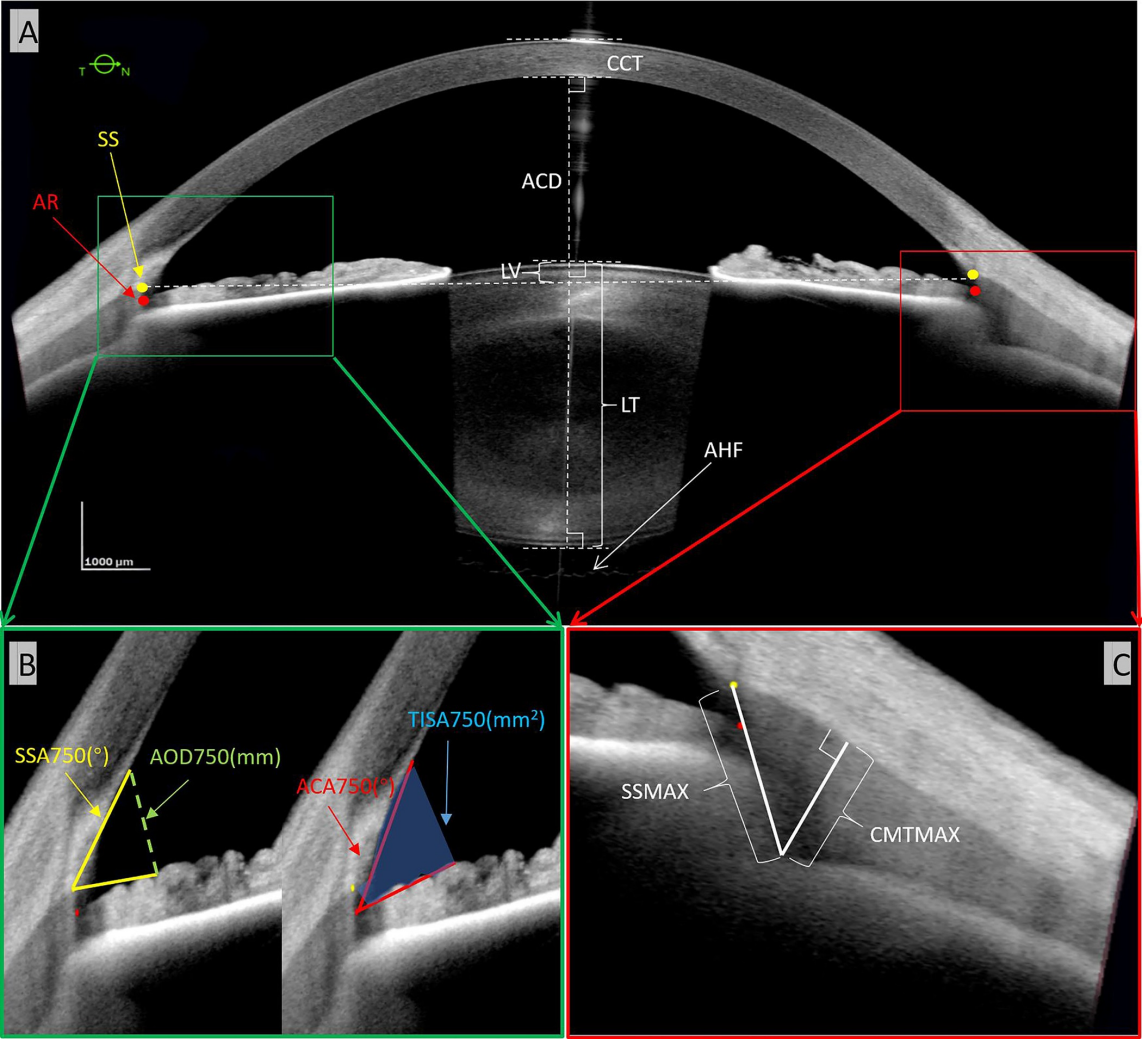
Anterior segment OCT endpoints. (A) The overall anterior ocular biometry with SS, scleral spur; AR, angle recess; CCT, central cornea thickness; AD, aqueous depth; PD, pupil diameter; LV, lens vault (the perpendicular distance between the anterior pole of the crystalline lens and the horizontal line joining the two scleral spurs); and LT, lens thickness. The undulating face of the anterior vitreous face is seen posterior to the lens (arrow). (B) Anterior angle parameters. AOD_750_ (mm), the perpendicular distance from a point on the posterior corneal surface that is 750 μm anterior to the scleral spur and anterior iris surface; ACA_750_(^o^), the angle at the conjunction of the line connecting the angle recess point to the AOD_750_ iris endpoint and the line connecting the angle recess point to the AOD_750_ corneal endpoint; SSA_750_(^o^), the angle at the conjunction of the line connecting the scleral spur to the AOD_750_ iris endpoint and the line connecting the scleral spur to the AOD_750_ corneal endpoint; TISA_750_ (mm^2^), the trapezoidal area defined by the AOD_750_, the inner corneo-scleral wall, and the perpendicular distance between scleral spur and iris. (C) Ciliary muscle parameters. CMTMAX, ciliary muscle thickness at the point of maximum thickness; SSMAX, distance between scleral spur and the thickest point of the ciliary muscle. Scale bars: 1000 μm.

AS-OCT images obtained along the horizontal meridian across the corneal vertex were selected for analyses. Measurements of pupil diameter, central corneal thickness (CCT), lens thickness, and aqueous depth were provided automatically by the device (Fig 1).

A semi-automated approach was necessary for angle measurements, lens vault, anterior chamber angle distance, and spur-to-spur distance (Fig 1). Investigators (XX and GC) had to first identify and place markers on the anterior chamber angle recess points and scleral spurs on both the temporal and nasal sides of the image. Subsequently, the software provided these measurements.

For the ciliary muscle, all measurements were fully manual. The first step was to manually segment the scleral-ciliary muscle tissue boundaries by extending the posterior cornea segmentation line using segmentation tools within the ANTERION. This line was deleted from the image to prevent bias during actual ciliary muscle thickness measurements. The swept-source AS-OCT device applies Snell’s Law using the relative indices of refraction for the anterior and posterior corneal surfaces and at the iris and lens surfaces at 1300 μm to correct the OCT image. Additionally, the changing speed of light within tissue is accounted for by appropriate scaling of sections of the A-scan according to the refractive index. Thus, by manually extending the segmentation line into the scleral-ciliary muscle border, the calipers were activated, and the software used the index of refraction relative to the anterior and posterior cornea surface to correct the OCT image in the correspondent areas. The scleral spur and angle recess were manually identified. Then, using previously published endpoints [21–30], the maximum ciliary muscle thickness (CMTMAX) defined as the distance of a line between the maximum ciliary muscle apex to the scleral-ciliary muscle border that is perpendicular to this border and the distance of a line between the scleral spur to the maximum ciliary muscle apex (SSMAX) were manually measured (Fig 1).

Repeatability assessment was conducted using a clinically competent masked grader (XX) who manually identified the angle recesses, scleral spurs, and made all ciliary muscle measurements in images acquired for repeatability purposes (n = 40; see above). For reproducibility, the same steps were performed twice using randomly assigned images (n = 40) either by the same investigator (XX; intra-observer) or by two investigators (XX and GC; inter-observer) who were also masked to the conditions.

### Assessment of averaging

The ANTERION has live acquisition eye tracking that uses the corneal LED reflexes, to center the B-scans on the corneal vertex. To study the effect of image averaging on ciliary muscle imaging and measurements, the Imaging Application was used since it allowed for adjusting scan pattern parameters, including the amount of averaging. This is different from the Metrics Application, which was used above. Because there is no automatic segmentation on the Imaging Application, there is no refraction correction, and measurements in microns is not possible. Instead, measurements were made in pixels. Nine subjects underwent imaging twice (back-to-back) with identical settings using an “Averaging Setting of 1” (no averaging) followed by a second scan with identical settings using an “Averaging Setting of 8” (averaging from 8 images, the maximum setting). Of 9 subject, 8 subjects had both eyes images, and one subject had one eye imaged (17 total eyes). Unlike biological studies where there may be interdependence between eyes and where only one eye is investigated, both eyes and ciliary muscles (nasal and temporal) were investigated as individual systems because the evaluation was solely in regard to the impact of the device averaging as opposed to studying a biological process. This resulted in a sample size of 34 pairs of images (17 X 2 sides of the eye) for “Averaging Setting of 1” and 34 pairs of images for “Averaging Setting of 8.” For all images, the CMTMAX and SSMAX were measured using in-built calipers by a grader masked to the condition.

### Statistical analysis

Descriptive statistics, such as mean and standard deviation or median and quartiles, range for continuous variables, and frequency and percentage for categorical variables, were calculated for demographic, biometric, and accommodative measures. Statistical analyses were performed using SPSS (SPSS for Windows, v. 26.0, IBM-SPSS, Chicago, IL). The normal distribution of continuous data was examined using the Shapiro–Wilk’s W test. Parameters with a normal distribution were presented as mean ± standard deviation. Parameters that did not follow a normal distribution were presented as median and quartiles. A paired two sample t-test was used to compare the mean difference for each biometric parameter between nasal and temporal measures within the same eye, such as ciliary muscle measurements (CMTMAX and SSMAX) and anterior chamber angle parameters (AOD_750_, TISA_750_, SSA_750_, and ACA_750_). Univariate and multivariable linear regression analyses were performed (independent variables: age and refractive status; dependent variables: the various anterior segment parameters). For parameters with both nasal and temporal measures, both sides were analyzed separately during the above linear regression analyses.

The MedCalc program (v. 11.5.1.0 for Windows; available at: www.medcalc.be; accessed April 20, 2011) was used to assess test-retest repeatability and intra- and inter-observer reliability of the anterior segment biometric parameters measurements. Intraclass correlation coefficient (ICC) was calculated to determine the degree of test-retest reliability, and the degree of inter- and intra-observer reproducibility. Bland–Altman analysis was calculated to determine the test-retest variabilities and inter- and intra-observer variabilities. It was also used to assess the impact of averaging on ciliary muscle measurements [31]. To yield a statistical measure, another approach was taken where the difference (or error) between the pairs of images were calculated for Averaging 1 and Averaging 8 and compared via a Wilcoxon ranked-sum test.

## Results

### Overall anterior segment OCT performance

Sixty-three right eyes were imaged from 63 subjects, and the demographics are displayed in Table 1. The averaged refraction spherical equivalent was -1.73 ± 2.0 diopters (D) (range: -8.25 to +3.50D). Horizontal images were obtained where the entire anterior chamber could be seen including the cornea, angle, iris, ciliary muscle, and phakic lens from anterior to posterior capsule (Fig 1A to 1C). Even the undulating anterior hyaloid face could be seen posterior to the lens (Fig 1A; arrow).

**Table 1:** Demographic, ophthalmologic characteristics of the study population (mean ± standard deviation).

	Study Population
No. of participants	63
No. of eyes	63
Age median quartiles (Year)	41, 32~57
Range	23–74
Gender (Male/Female)	36/27
Spherical equivalent refraction (Diopter)	-1.73 ± 2.0
Range	-8.25 ~ +3.5

Looking at the repeatability, excellent intraclass correlations (ICC) were noted (0.90–1.00) for all assessments (Table 2). Looking at the ICC 95% limits of agreement, higher repeatability was generally seen comparing automatic compared to semi-automatic to manual endpoints.

**Table 2:** Test-retest repeatability analyses.

Test	Inter-test	
	Difference (95% LoA)	ICC (95% CI)
**Biometric Parameters of Ciliary Muscle by Manual Method**		
CMTMAX (μm)	-10.0 (-154.8–134.8)	0.92(0.88–0.97)
SSMAX (μm)	12.1 (-143.7–171.75)	0.90(0.75–0.95)
**Biometric Parameters by Semi-Automatic Method**		
Spur-to-Spur Distance (mm)	0.08 (-0.64–0.79)	0.94 (0.91–0.96)
ACA Distance (Mm)	-0.02(-0.13–0.13)	0.98 (0.98–0.99)
Lens Vault (Mm)	0.00(-0.06–0.07)	0.99 (0.99–1.00)
ACA _750_°)	-0.05 (-8.80–7.90)	0.94 (0.91–0.96)
SSA _750_°)	-0.02 (-4.08–4.05)	0.94 (0.91–0.96)
AOD _750_(mm)	-0.03 (-0.24–0.20)	0.92 (0.87–0.93)
TISA _750_ (mm^2^)	-0.01 (-0.10–0.10)	0.92 (0.88–0.95)
**Biometric Parameters by Automatic Method**		
CCT (μm)	-0.1 (-2.70–2.50)	1.00 (1.00–1.00)
Lens Thickness (mm)	0.00 (-0.02–0.03)	1.00 (1.00–1.00)
Aqueous Depth (mm)	-0.00(-0.03–0.03)	1.00 (1.00–1.00)

LoA: limits of agreement. CI: confidence intervals.

The intraclass correlation coefficient (ICC) was calculated to determine the degree of test-retest reliability. ICC values less than 0.5 are indicative of poor reliability, values between 0.5 and 0.75 indicate moderate reliability, values between 0.75 and 0.9 indicate good reliability, and values greater than 0.90 indicate excellent reliability. The test-retest variabilities were determined by Bland–Altman analysis. The test-retest reproducibility was analyzed based upon 40 eyes.

Inter- and intra-observer reproducibilities were determined for the endpoints where some manual step was required for the measurement (Table 3). The 95% limits of agreement of the mean differences suggested that the intra-observer agreement was superior to the inter-observer agreement. Otherwise, high intra-observer reproducibility with ICC values between 0.93–1.00 and high inter-observer reproducibility with ICC values of 0.88–1.00 were found (Table 3).

**Table 3:** Inter-observer and intra-observer reproducibility analyses.

Measurement	Inter-observer		Intra-observer	
	Difference (95% LoA)	ICC (95% CI)	Difference (95% LoA)	ICC (95% CI)
CMTMAX (μm)	10.51 (-74.7–96.0)	0.95 (0.89–0.97)	-1.22 (-89.5–87.2)	0.96 (0.92–0.98)
SSMAX (μm)	1.34 (-134.5–137.2)	0.88 (0.77–0.94)	-5.85 (-106.8–94.9)	0.93 (0.87–0.96)
ACA distance (mm)	-0.01 (-0.1–0.08)	1.00 (0.99–1.00)	0.01 (-0.07–0.10)	1.00 (0.99–1.00)
Spur-to-spur distance (mm)	0.00 (-0.13–0.13)	0.99 (0.98–0.99)	0.02 (-0.11–0.14)	0.99 (0.98–0.99)
ACA _750_°)	0.05 (-5.5–5.6)	0.96 (0.92–0.98)	-0.3 (-6.6–6.0)	0.95 (0.91–0.97)
SSA _750_°)	-0.03 (-6.7–6.1)	0.93 (0.87–0.95)	-0.5 (-4.5–3.6)	0.96 (0.94–0.99)
AOD _750_ (mm)	-0.01 (-0.13–0.11)	0.94 (0.90–0.99)	-0.01 (-0.09–0.07)	0.96 (0.95–0.98)
TISA _750_ (mm^2^)	-0.01 (-0.04–0.04)	0.99 (0.97–1.00)	-0.01 (-0.04–0.03)	0.98 (0.96–1.09)
Lens vault (mm)	0.01 (-0.03–0.04)	0.99 (0.99–1.00)	0.00 (-0.04–0.04)	0.99 (0.98–1.00)

CMTMAX: Ciliary muscle thickness at the point of maximum thickness

SSMAX: The distance between scleral spur and the thickest point of the ciliary muscle

LoA: limits of agreement. CI: confidence intervals.

The intraclass correlation coefficient (ICC) was calculated to determine the degree of inter- and intro- observer reliabilities. ICC values less than 0.5 are indicative of poor reliability, values between 0.5 and 0.75 indicate moderate reliability, values between 0.75 and 0.9 indicate good reliability, and values greater than 0.90 indicate excellent reliability. The inter- and intro- observer variabilities were determined by Bland–Altman analysis. The inter-observer and intra-observer reliability were analyzed based upon 40 eyes.

### Impact of image averaging

**Fig 2:**
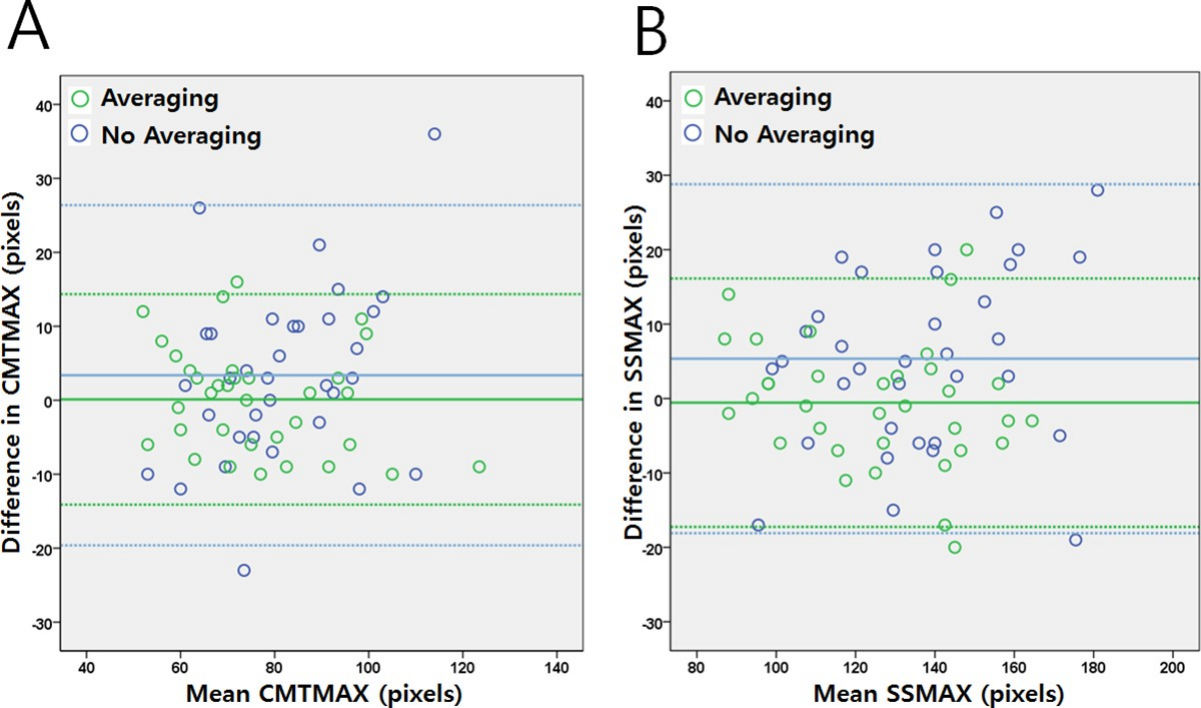
Impact of image averaging on ciliary muscle metrics. **(A)** Bland-Altman plots for CMTMAX comparing the differences between two images taken with (green; Averaging setting of 8) and without (blue; Averaging setting of 1) averaging. Without averaging (3.38 [-19.6, 26.3] pixels; bias line [95 LOA]) and with averaging (0.12 [-14.1, 14.3]). **(B)** Bland-Altman plots for SSMAX comparing the differences between two images taken with (green; Averaging setting of 8) and without (blue; Averaging setting of 1) averaging. Without averaging (5.35 [-18.1, 28.8] pixels; bias line [95 LOA]) and with averaging (-0.6 [-17.3, 16.1]). Note that during higher averaging, bias lines are closer to zero with narrower 95% limits of agreement areas for both CMTMAX and SSMAX. Solid lines = bias line. Dotted lines = positive and negative bounds for 95% limits of agreement (LOA).

A separate analysis was performed to assess the impact of image averaging on the ciliary muscle measurements. This was focused on the ciliary muscle given its deep position which makes OCT imaging difficult. The difference in CMTMAX and SSMAX measurements were determined from two images taken with an Averaging Setting of 1 and then at 8. Greater averaging resulted in a smaller difference that was statistically significant (CMTMAX; [Avg1: 9.3±7.4 pixels; Avg8: 5.9±4.0 pixels; Wilcoxon rank sum test *p* = 0.02]) (SSMAX; [Avg1: 10.8±7.2 pixels; Avg8: 6.2±5.1 pixels; Wilcoxon rank sum test *p* = 0.008]). Bland-Altman analyses were also performed for CMTMAX and SSMAX showing a tighter 95% limits of agreement comparing Averaging 8 compared to Averaging 1 and center bias lines closer to zero for higher averaging (Fig 2).

### Relationship of anterior segment parameters to age and refractive status

Subjects were recruited based on a distribution of ages. Given the attention to age, it was noted that younger volunteers were more likely to be myopic compared to older volunteers. Statistically, there was significant covariation between age and refractive status (R^2^ = 0.18, *p* = 0.001). Thus, univariate linear regressions were first conducted to evaluate the relationship between different anterior segment biometric parameters (such as ciliary muscle metrics, aqueous depth, ACA parameters, lens thickness and vault, and pupil size) to age and refractive status. Then, a multivariable linear regression analysis was carried out to further clarify the effects of age and refractive status on each of the above biometric parameters. Discrepancies between the results of univariate and multivariable analyses were likely due to the effect of variable co-variation in the univariate analyses.

### Ciliary muscle measurements

**Fig 3:**
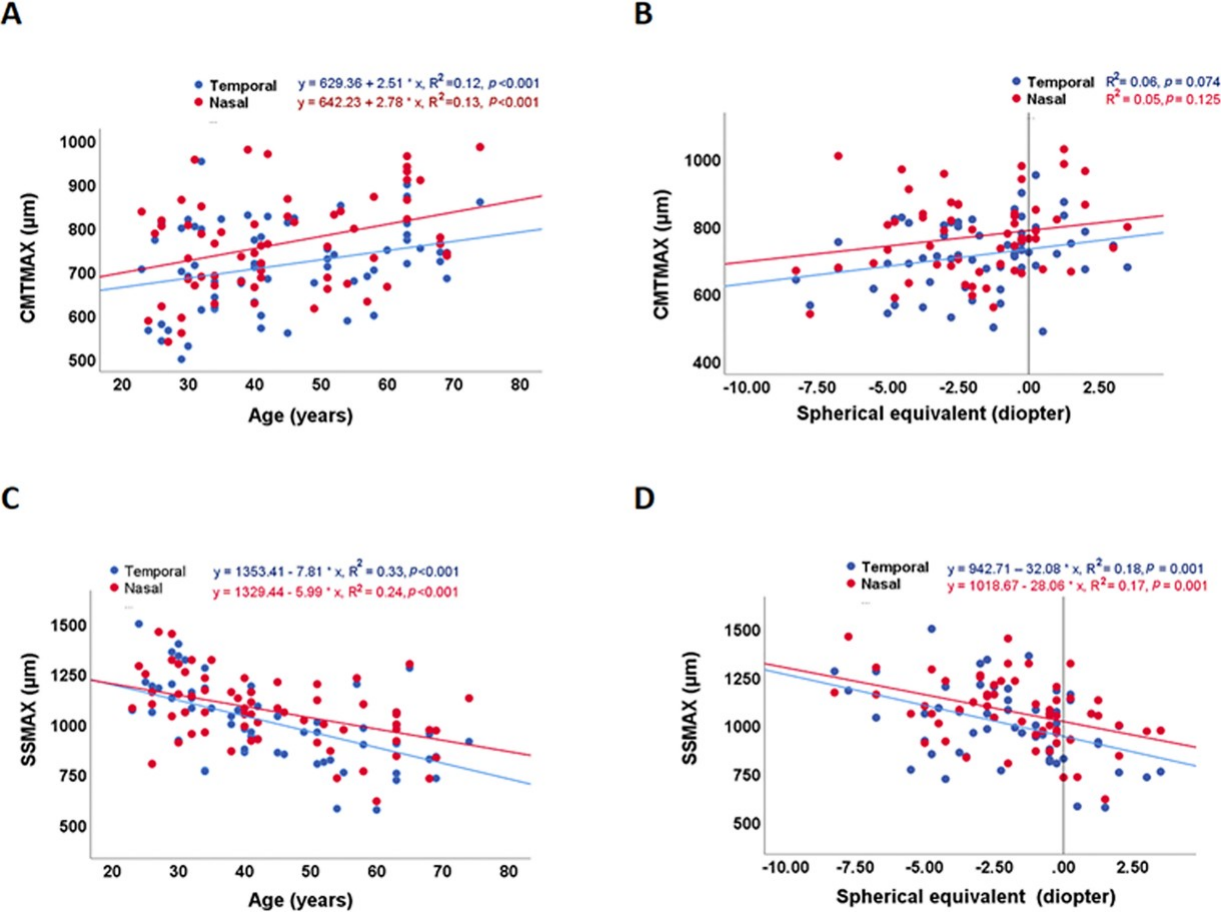
Univariate scatter plot comparison of ciliary muscle metrics versus age and spherical equivalent refractive error. **(A)** CMTMAX versus age. Both nasal and temporal CMTMAX showed significant positive linear relationships with age. (**B)** CMTMAX versus spherical equivalent refractive error. While qualitative trends of increased nasal and temporal CMTMAXs with increased spherical equivalent were seen, no statistical significances were found. (**C).** SSMAX versus age. Both nasal and temporal SSMAX show significant negative linear relationships with age. (**D).** SSMAX versus spherical equivalent. Both nasal and temporal SSMAX showed significant negative linear relationships with increasing hyperopic refractive error. CMTMAX: Ciliary muscle thickness at point of maximum thickness; SSMAX: Distance between scleral spur and the thickest point of the ciliary muscle.

The mean values of CMTMAX and SSMAX on the nasal side were significantly greater than those on the temporal side (Table 4; *p*<0.001 and *p* = 0.006, respectively). In terms of CMTMAX, univariate analysis showed positive linear relationships between both nasal and temporal values to age (Fig 3A; nasal R^2^ = 0.13, *p*<0.001; temporal R^2^ = 0.12, *p*<0.001). For univariate analysis against refractive status, there was no statistically significant relationship (Fig 3B; nasal R^2^ = 0.05, *p*<0.125; temporal R^2^ = 0.06, *p*<0.07). Multivariable analyses linear regression analyses confirmed these results (Table 5; age *p*<0.001–0.004 and refractive status *p* = 0.22–0.84). The above results suggested that the nasal and temporal CMTMAX sized increased with increasing age.

**Table 4:** Biometric parameters of ciliary muscle and anterior segment of adult human eyes.

Biometric Parameters	Mean Values (Mean ± Standard Deviation)		*p*-Value
**Ciliary Muscle Biometrics**	Nasal	Temporal	
CMTMAX (μm)	765.10 ± 116.26	715.17 ± 101.44	<0.001
SSMAX (μm)	1056.48 ± 173.94	1008.35 ± 194.04	0.006
**CCT** (μm)	547.56 ± 34.42		
**Anterior Chamber**			
Aqueous Depth (mm)	2.97 ± 0.37		
ACA Distance (mm)	12.13 ± 0.37		
Spur-to-spur Distance (mm)	12.13 ± 0.36		
**Anterior Chamber Angle**	Nasal	Temporal	
ACA _750_ (°)	38.12 ± 13.67	37.01 ± 12.27	0.24
SSA _750_ (°)	40.38 ± 14.72	39.35 ± 12.23	0.32
AOD _750_ (mm)	0.65 ± 0.35	0.66 ± 0.28	0.33
TISA _750_ (mm^2^)	0.33 ± 0.17	0.32 ± 0.15	0.10
**Lens**			
Lens Thickness (mm)	4.27 ± 0.41		
Lens Vault (mm)	0.25 ± 0.33		
**Pupil Diameter** (mm)	5.42 ± 1.09		

CMTMAX: Ciliary muscle thickness at point of maximum thickness

SSMAX: Distance between scleral spur and the thickest point of the ciliary muscle

CCT: Central cornea thickness

ACA distance: The distance between two anterior chamber angles

ACA _750_: Anterior chamber angle measured at 750 μm from the scleral spur

SSA _750_: Scleral spur angle measured at 750 μm from the scleral spur

AOD _750_: Angle-opening distance measured at 750 μm from the scleral spur

TISA _750_: Trabecular-iris space area measured at 750 μm from the scleral spur

*p* value: statistical significance of the difference between temporal and nasal aspects at the same measurement location.

**Table 5:** Multivariable linear regression analysis of the relationship between age and spherical equivalent refractive error to ciliary muscle metrics.

Dependent Variable		R^2^	p*	Independent variable	Non-standardized Beta (95% CI)	Standardized Beta	*p*†
CMTMAX	Nasal	0.13	0.004	Age	2.78 (0.92~4.65)	0.36	0.004
				Refractive Error	/	/	0.84
	Temporal	0.11	<0.001	Age	2.47 (1.21~3.73)	0.33	<0.001
				Refractive Error	/	/	0.22
SSMAX	Nasal	0.26	<0.001	Age	-4.32 (-7.31~-1.32)	-0.36	0.01
				Refractive Error	-18.01 (-34.84~-1.19)	-0.27	0.03
	Temporal	0.31	<0.001	Age	-5.23 (-7.41~-3.06)	-0.41	<0.001
				Refractive Error	-17.89 (-30.11~-5.66)	-0.25	0.005

p* for multivariable linear regression models, and *p*† for independent variable

In terms of SSMAX, in univariate analysis, both nasal and temporal values showed negative linear relationships with age (Fig 3C; nasal R^2^ = 0.24, *p*<0.001; temporal R^2^ = 0.33, *p*<0.001) and more hyperopic refractive status (Fig 3D; nasal R^2^ = 0.17, *p* = 0.001; temporal R^2^ = 0.18, *p* = 0.001). In multivariable linear regression analysis, both nasal and temporal SSMAX still showed independent negative linear relationships with age (Table 5; *p*<0.001–0.01) and more hyperopic refractive status (Table 5; *p* = 0.005–0.03). These results suggested that the ciliary muscle apex was positioned more anteriorly in older and more hyperopic subjects.

### Lens thickness and lens vault

For lens thickness, univariate analysis showed a positive linear relationship with age (S1A Fig; R^2^ = 0.61, *p*<0.001), and a positive linear relationship with refractive status (S1B Fig; R^2^ = 0.17, *p* = 0.001). Multivariable linear regression analysis showed that increased age was still related to a thicker crystalline lens (Table 6; *p*<0.01), whereas the lens thickness was no longer significantly associated with refractive status (Table 6; *p* = 0.24). These results suggested that age had an impact upon lens thickness while refractive status did not.

**Table 6:** Multivariable linear regression analyses of the relationships between age and spherical equivalent refractive error to lens, aqueous depth, and pupil diameter.

Dependent variable	R^2^	p*	Independent variable	Non-standardized Beta (95% CI)	Standardized Beta	*p*†
Lens Thickness	0.61	<0.01	Age	0.02 (0.02~0.03)	0.73	<0.01
			Refractive Error	/	/	0.24
Lens Vault	0.47	<0.01	Age	0.01 (0.01~0.02)	0.43	<0.01
			Refractive Error	0.05 (0.02~-0.08)	0.39	<0.01
Aqueous Depth	0.36	<0.01	Age	-0.01 (-0.02~-0.00)	-0.38	0.01
			Refractive Error	-0.05 (-0.08~-0.01)	-0.33	0.01
Pupil Diameter	0.21	<0.01	Age	-0.35 (-0.53~-0.18)	-0.46	<0.01
			Refractive Error	/	/	0.83

p* for multivariable linear regression models, and *p*† for independent variable

As for lens vault, univariate analysis showed a positive linear relationship with age (S1C Fig; R^2^ = 0.35, *p*<0.001), and a positive linear relationship with refractive status (S1D Fig; R^2^ = 0.31, *p*<0.001). Multivariable linear regression analysis confirmed that lens vault was independently and significantly associated with both age (Table 6; *p*<0.01) and more hyperopic refractive status (Table 6; *p*<0.01).

### Angle measurements

Eight anterior chamber angle biometric endpoints were analyzed: anterior chamber angle (ACA_750_), scleral spur angle (SSA_750_), angle-opening distance (AOD_750_), and trabecular-iris space area (TISA_750_) on the temporal and nasal sides (Fig 1). There were no statistically significant differences comparing both sides (Table 4; *p* = 0.1–0.33).

Univariate analyses showed a negative linear relationship between all of these end-points with increased age and more hyperopic refractive status (S2A to S2H Fig; R^2^ = 0.17–0.32, *p*<0.001–0.003). Multivariable linear regression analyses confirmed the results of univariate analyses showing that there were independent and significant negative linear relationships between all angle metrics on both nasal and temporal sides with increased age and more hyperopic refractive status (Table 7; *p*<0.001–0.03).

**Table 7:** Multivariable linear regression analysis of the relationship between age and spherical equivalent refractive error to anterior chamber angle dimensions.

Dependent Variable		R^2^	*F*	p*	Independent variable	Non-standardized Beta (95% CI)	Standardized Beta	*t*	*p*†
ACA_750_	Temporal	0.44	22.54	<0.01	Age	-0.43 (-0.66~-0.20)	-0.40	-3.69	<0.01
					Refractive Error	-2.39 (-3.70~-1.08)	-0.40	-3.66	0.01
	Nasal	0.40	18.91	<0.01	Age	-0.25 (-0.47~-0.04)	-0.27	-2.28	0.03
					Refractive Error	-2.65 (-3.88~-1.41)	-0.48	-4.29	0.01
SSA_750_	Temporal	0.42	21.00	<0.01	Age	-0.44 (-0.68~-0.20)	-0.40	-3.68	0.01
					Refractive Error	-2.30 (-3.65~-0.95)	-0.37	-3.41	0.01
	Nasal	0.35	15.07	<0.01	Age	-0.25 (-0.47~-0.04)	-0.27	-2.28	0.03
					Refractive Error	-2.26 (-3.49~-1.03)	-0.43	-3.67	0.01
AOD_750_	Temporal	0.32	13.76	<0.01	Age	-0.01 (-0.02~-0.00)	-0.38	-3.23	0.02
					Refractive Error	-0.06 (-0.10~-0.02)	-0.30	-2.50	<0.01
	Nasal	0.39	18.30	<0.001	Age	-0.01 (-0.01~-0.00)	-0.25	-2.27	0.03
					Refractive Error	-0.06 (-0.08~-0.03)	-0.48	-4.32	0.01
TISA_750_	Temporal	0.32	11.45	<0.01	Age	-0.01 (-0.01~-0.00)	-0.39	-3.33	<0.01
					Refractive Error	-0.02 (-0.03~-0.00)	-0.29	-2.36	<0.01
	Nasal	0.32	12.64	<0.01	Age	-0.00 (-0.00~0.00)	-0.32	-3.74	<0.001
					Refractive Error	-0.02 (-0.04~-0.01)	-0.37	-4.44	<0.001

p* for multivariable linear regression models, and *p*† for independent variable

### Other significant relationships (aqueous depth and pupil diameter)

For aqueous depth, univariate analyses showed negative linear relationships with age (S3A Fig; R^2^ = 0.27, *p*<0.001) and more hyperopic refractive status (S3B Fig; R^2^ = 0.24, *p*<0.001). The result of multivariable linear regression analyses was consistent with the result of univariate analyses showing significant and independent relationships between decreased aqueous depth and increased age (Table 6; *p* = 0.01) and more hyperopic refractive status (Table 6; *p* = 0.01).

For pupil diameter, univariate analysis showed negative linear relationships between pupil diameter and age (S4A Fig; R^2^ = 0.21, *p*<0.001), while there was no relationship with refractive error (S4B Fig; R^2^ = 0.001, *p* = 0.81). Multivariable linear regression analyses confirmed the above results showing a positive linear relationship with age (Table 6; *p*<0.01) but not refractive status (Table 6; *p* = 0.83).

### Non-significant relationships (central corneal thickness [CCT], spur-to-spur distance, and anterior chamber angle [ACA] distance)

Not every measured endpoint demonstrated a statistically significant relationship to age or refractive status in univariate analyses. Mean CCT did not correlate with age (R^2^ = 0.09, *p* = 0.40) or refractive status (R^2^ = 0.03, *p* = 0.81). Mean ACA distance did not correlate with age (R^2^ = 0.06, *p* = 0.14) or refractive status (R^2^ = 0.04, *p* = 0.45). Mean spur-to-spur distance did not correlate with age (R^2^ = 0.03, *p* = 0.40) or refractive status (R^2^ = 0.03, *p* = 0.46).

## Discussion

High quality anterior segment images were obtained using a new swept-source AS-OCT. From single images, the entire anterior segment could be visualized from the cornea to the posterior lens and ciliary body on both nasal and temporal quadrants. Thus, many anterior segment parameters could be studied simultaneously. Measurements obtained from these images were a combination of automated, semi-automated, and manual. In all cases, consistent repeatability was determined for all measurements. For metrics that required manual steps, good reproducibilities between graders were observed as well. Image averaging improved the ability to measure ciliary muscle parameters. Thus, swept-source AS-OCT with tracking is a robust approach that can simultaneously assess anterior segment structural changes across many parameters which may be useful for better studying normal physiology and disease.

Demonstrating repeatable and reproducible imaging with this new diagnostic device is a key outcome. Furthermore, most of the individual findings support previous results in the literature. This includes the ciliary muscle getting larger with age [21, 27] in addition to other associations between the lens, anterior chamber angle, and pupil to age or refractive status [26, 29, 32–37]. Ultimately, obtaining repeatable and reproducible measurements using a new swept-source AS-OCT that also accounts for known anatomical alterations gives future users confidence in using this device to study biology as well as potentially diagnosing and treating disease.

Image tracking with averaging is a unique aspect of this swept-source AS-OCT as well. For sweptsource OCT, other anterior segment OCTs have been developed [11–17], but they lacked image tracking and averaging and could not visualize the entire lens thickness (from anterior to posterior pole). The importance of averaging is the main reason why posterior segment OCT is so widely used and clinically applicable [6–8]. In this report, less error was seen in ciliary muscle measurements when images were taken with high averaging. Thus, for the anterior segment, averaging is important to visualize difficult to image structures, like the ciliary muscle. Combining longer wavelength swept-source imaging with image tracking and averaging allows for excellent simultaneous imaging of deep structures such as the ciliary muscle and entire lens thickness.

Overall, improved imaging accuracy and capability is important to better understand anterior segment physiology and disease. For example, better deep imaging of the lens can provide information about the posterior capsule to help distinguish posterior polar cataracts from posterior subcapsular cataracts [38]. Seeing the absence or presence of an intact posterior capsule prior to surgery significantly impacts surgical planning. Also, visualizing the entire lens and ciliary muscle in one image is important to understand accommodation. Presbyopia is the age-related loss of accommodation [39]. Prior accommodation research utilized different modalities (OCT [16, 21, 22, 24, 25, 30, 32, 40], ultrasound biomicroscopy [41], or MRI [42, 43]) or variations of the same modality (combining different types of custom-built OCTs [23, 44] to image different parts of the eye). Together, this precludes simultaneous assessment of all anterior segment anatomical alterations during accommodation before and after presbyopia, contributing to the challenge of understanding presbyopia pathophysiology. Lastly, imaging deep in the eye may have potential utility in glaucoma. Note that even the anterior hyaloid face can be seen using this swept-source AS-OCT (Fig 1A). Malignant glaucoma is a narrow angle glaucoma that also has competing pathophysiological hypotheses [45]. The anterior hyaloid face is generally thought to be important, and vitrectomy should relieve and prevent malignant glaucoma. However, malignant glaucoma can recur after vitrectomy, and it has been proposed [46] that this is due to the failure to disrupt the anterior hyaloid face during surgery. Now, with improved deep imaging, the success of anterior hyaloid removal can also be assessed.

Several limitations must also be discussed for this work. First, the distribution of subjects was relatively normally distributed across ages. However, it was not simultaneously normally distributed across age and refractive status. To achieve this, a much large sample size is needed. Also, the advantages of image tracking were not fully realized. Again, tracking allows for two benefits. First, tracking allows for image averaging to improve signal-to-noise ratio [7, 8]. This was demonstrated here for a deep anatomical structure such as the ciliary muscle. However, tracking also permits following OCT B-scans longitudinally over time in the location of the same subject. This allows for better studying the natural history of disease [6] and monitoring the impact of disease treatment. Given that the tracking for this swept-source AS-OCT relies upon arranging the B-scans based upon the corneal light reflex pattern, it is difficult to position a subject across different days in the exact same position to reproducibly obtain the exact same light reflex pattern. Thus, tracking for the purpose of setting a reference image for longitudinal imaging in the same location over time is currently difficult and still needs to be studied for this device. Alternative future options include tracking based on OCT landmarks, such as the scleral spur, using machine-based learning identification [46].

In conclusion, we describe a new swept-source AS-OCT device with eye tracking and image averaging. We demonstrate the ability to assess superficial and deep anterior segment structures, including the ciliary body and the entire lens thickness, simultaneously from single images in a repeatable and reproducible fashion. These advances can improve the clinical utility of AS-OCT, and additional research is needed to determine the clinical significance of these measurements.

## Supporting information

S1 Checklist (DOCX)

S1 Fig Univariate scatter plot comparison of lens metrics versus age and spherical equivalent refractive error. **(A)** Lens thickness versus age. **(B)** Lens thickness versus spherical equivalent refractive error. **(C)** Lens vault versus age. **(D)** Lens vault versus spherical equivalent refractive error.
(TIFF)

S2 Fig Univariate scatter plot comparison of anterior chamber angle metrics versus age and spherical equivalent refractive error. **(A)** Nasal and temporal ACA_750_ versus age. **(B)** Nasal and temporal ACA_750_ versus spherical equivalent refractive error. **(C)** Nasal and temporal SSA_750_ versus age. **(D)** Nasal and temporal SSA_750_ versus spherical equivalent refractive error. **(E)** Nasal and temporal AOD_750_ versus age. **(F)** Nasal and temporal AOD_750_ versus spherical equivalent refractive error. **(G)** Nasal and temporal TISA_750_ versus age. **(H)** Nasal and temporal TISA_750_ versus spherical equivalent refractive error.
(TIFF)

S3 Fig Univariate scatter plot comparison of aqueous depth versus age and spherical equivalent refractive error. **(A)** Aqueous depth versus age. **(B)** Aqueous Depth versus spherical equivalent refractive error.
(TIFF)

S4 Fig Univariate scatter plot comparison of pupil diameter versus age and spherical equivalent refractive error. **(A)** Pupil diameter versus age. **(B)** Pupil diameter versus spherical equivalent refractive error.
(TIFF)

S1 Data (XLSX)
