# Supplementary figures and images for: Age- and refraction-related changes in anterior segment anatomical structures measured by swept-source anterior segment OCT
Source: PLoS One. 2020 Oct 23;15(10):e0240110. doi: 10.1371/journal.pone.0240110 (PMC7584205; doi:10.1371/journal.pone.0240110)

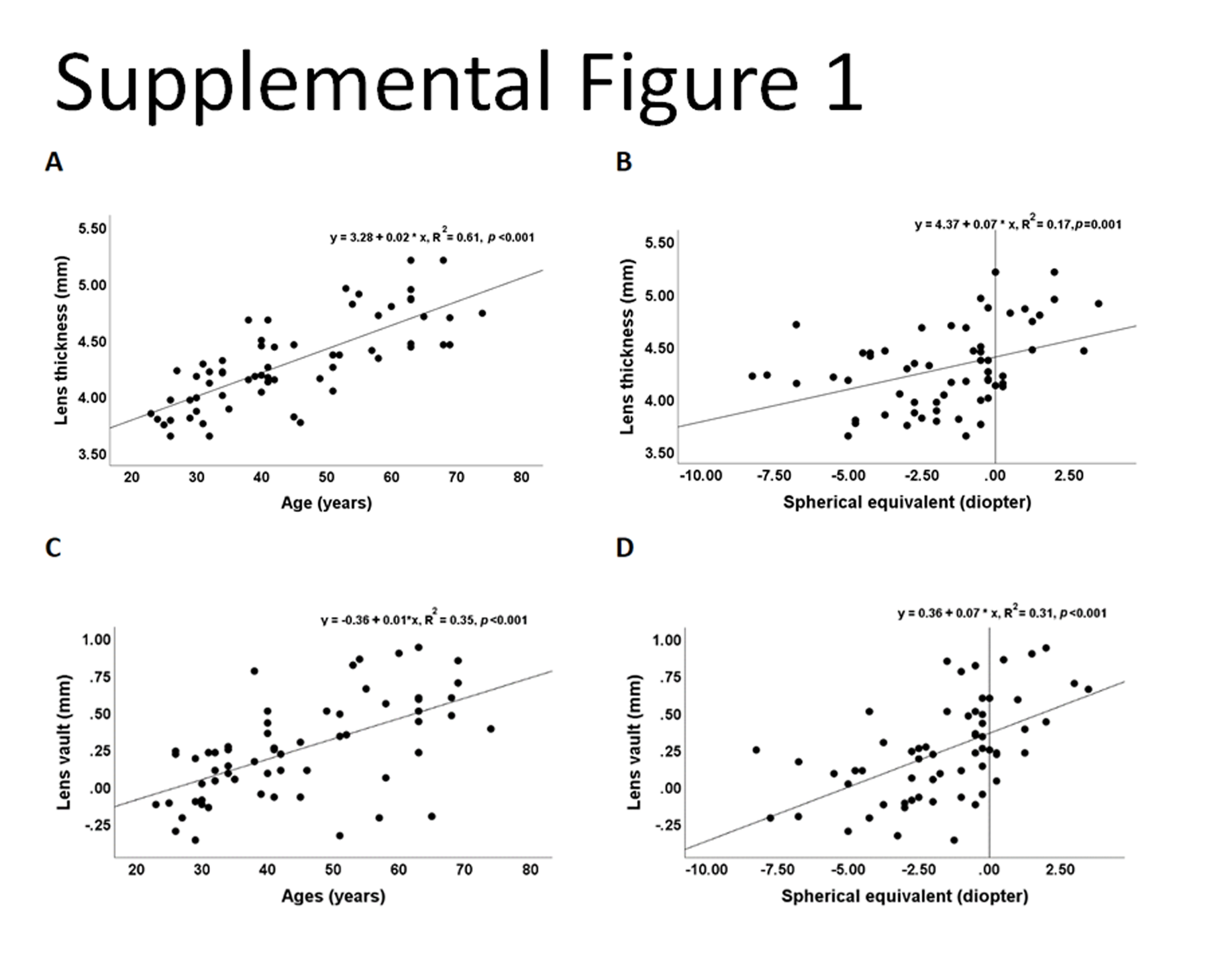

Supplement: S1 Fig — (A) Lens thickness versus age. (B) Lens thickness versus spherical equivalent refractive error. (C) Lens vault versus age. (D) Lens vault versus spherical equivalent refractive error. (TIFF) [file pone.0240110.s002.tiff]

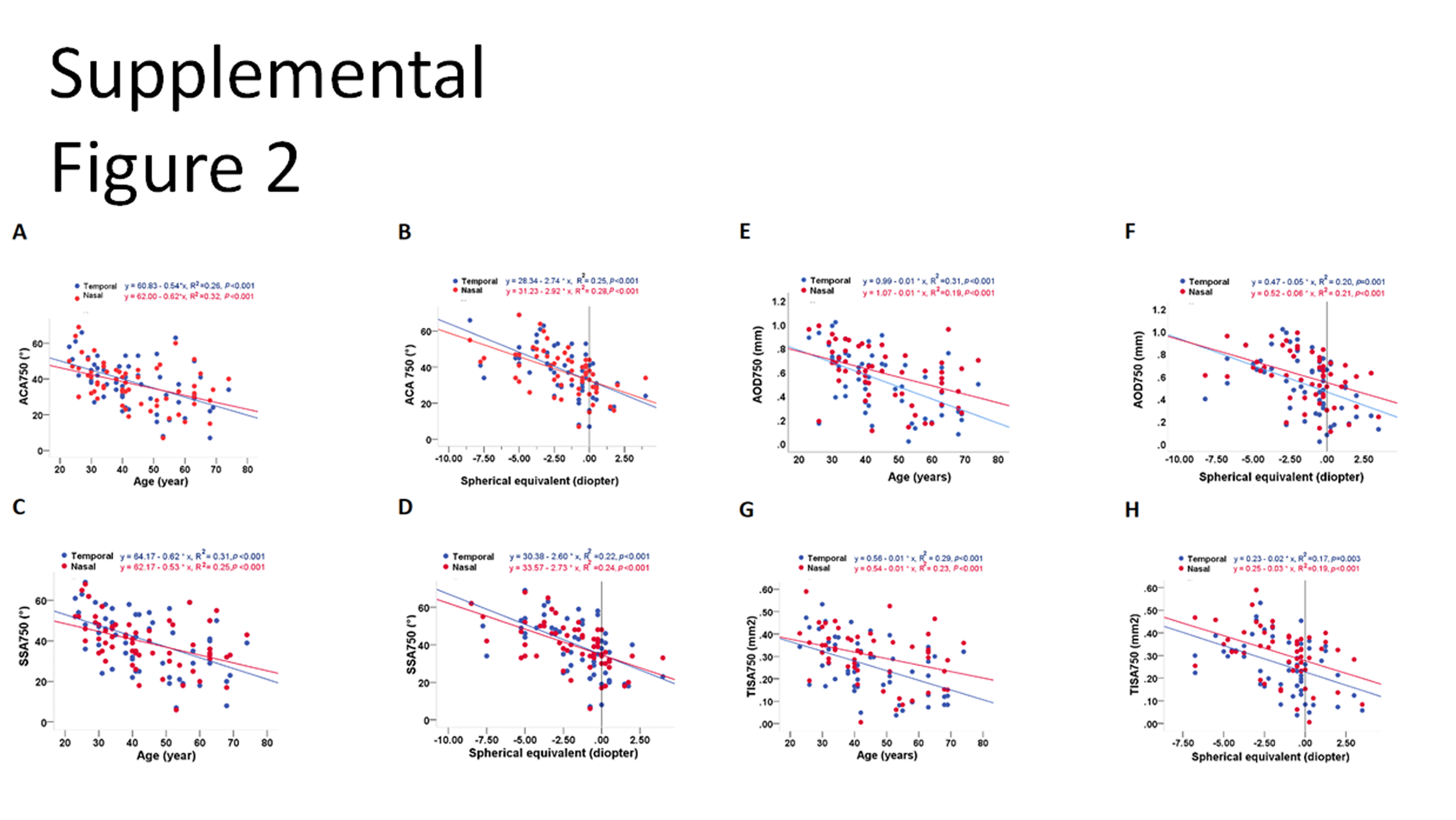

Supplement: S2 Fig — (A) Nasal and temporal ACA750 versus age. (B) Nasal and temporal ACA750 versus spherical equivalent refractive error. (C) Nasal and temporal SSA750 versus age. (D) Nasal and temporal SSA750 versus spherical equivalent refractive error. (E) Nasal and temporal AOD750 versus age. (F) Nasal and temporal AOD750 versus spherical equivalent refractive error. (G) Nasal and temporal TISA750 versus age. (H) Nasal and temporal TISA750 versus spherical equivalent refractive error. (TIFF) [file pone.0240110.s003.tiff]

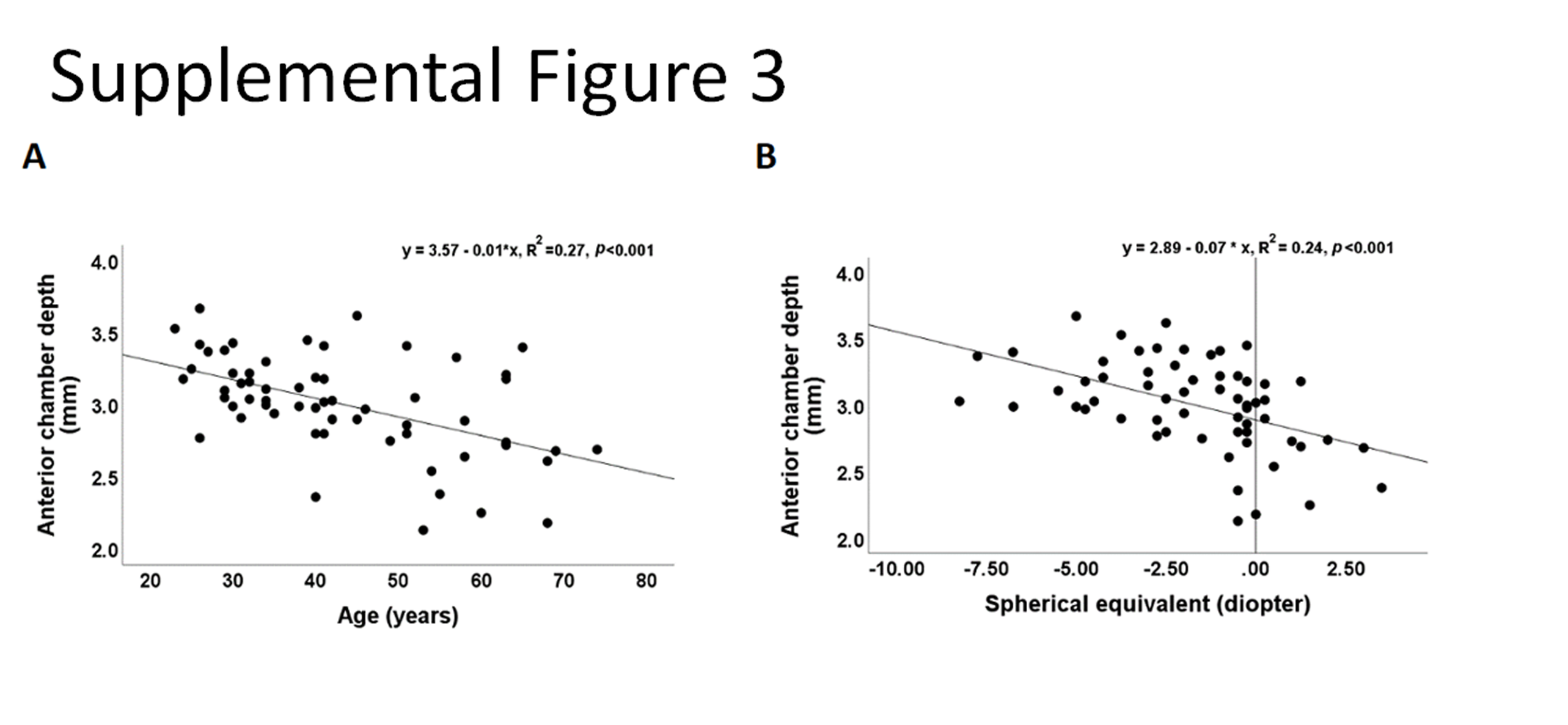

Supplement: S3 Fig — (A) Aqueous depth versus age. (B) Aqueous Depth versus spherical equivalent refractive error. (TIFF) [file pone.0240110.s004.tiff]

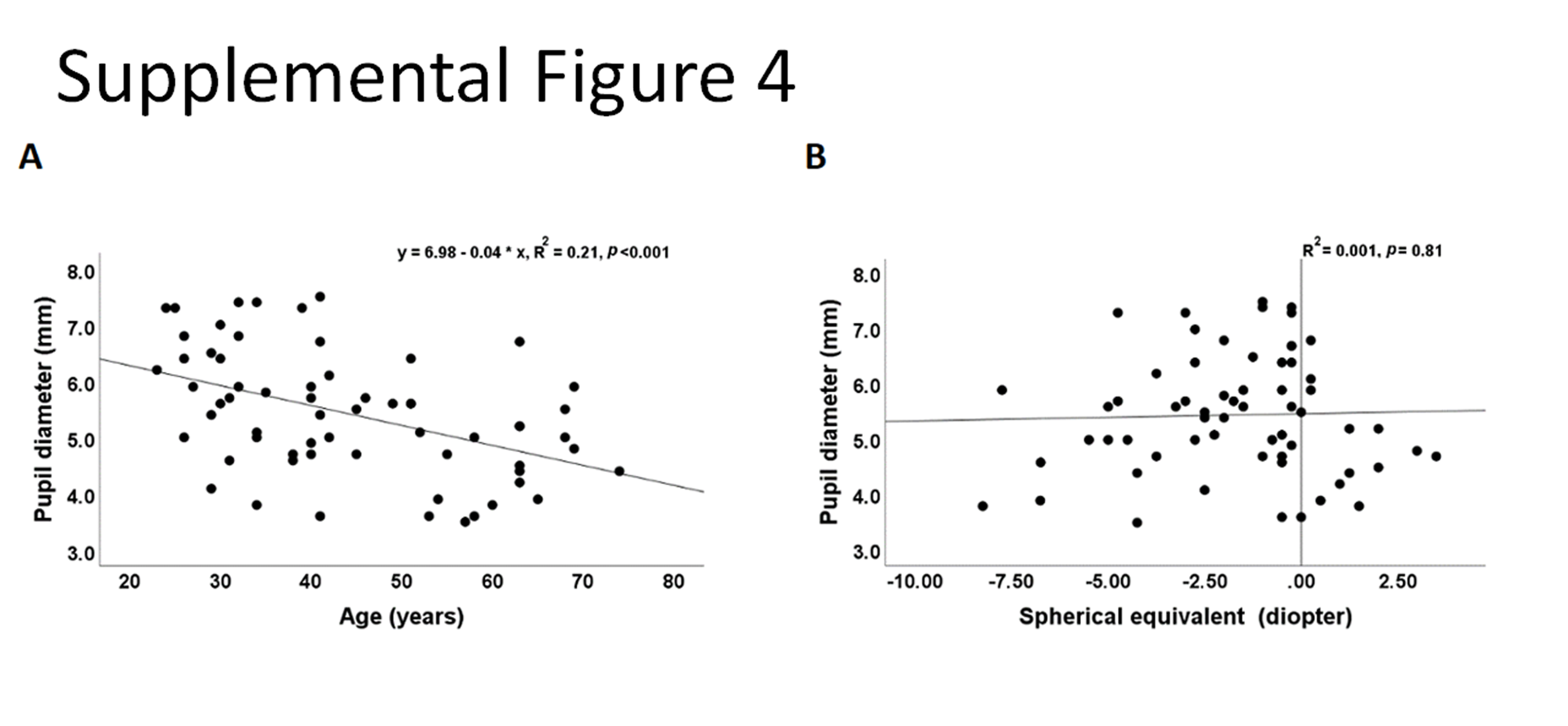

Supplement: S4 Fig — (A) Pupil diameter versus age. (B) Pupil diameter versus spherical equivalent refractive error. (TIFF) [file pone.0240110.s005.tiff]
